# Detecting circular RNA from high-throughput sequence data with de Bruijn graph

**DOI:** 10.1186/s12864-019-6154-7

**Published:** 2020-03-05

**Authors:** Xin Li, Yufeng Wu

**Affiliations:** Department of Computer Science and Engineering, University of Connecticut, Storrs, 06269 CT USA

**Keywords:** Circular RNA, High-throughput sequencing, Genomics, RNA-Seq, de Bruijn graph

## Abstract

**Background:**

Circular RNA is a type of non-coding RNA, which has a circular structure. Many circular RNAs are stable and contain exons, but are not translated into proteins. Circular RNA has important functions in gene regulation and plays an important role in some human diseases. Several biological methods, such as RNase R treatment, have been developed to identify circular RNA. Multiple bioinformatics tools have also been developed for circular RNA detection with high-throughput sequence data.

**Results:**

In this paper, we present circDBG, a new method for circular RNA detection with de Bruijn graph. We conduct various experiments to evaluate the performance of CircDBG based on both simulated and real data. Our results show that CircDBG finds more reliable circRNA with low bias, has more efficiency in running time, and performs better in balancing accuracy and sensitivity than existing methods. As a byproduct, we also introduce a new method to classify circular RNAs based on reads alignment. Finally, we report a potential chimeric circular RNA that is found by CircDBG based on real sequence data. CircDBG can be downloaded from https://github.com/lxwgcool/CircDBG.

**Conclusions:**

We develop a new method called CircDBG for circular RNA detection, which is based on de Bruijn graph. We conduct extensive experiments and demonstrate CircDBG outperforms existing tools, especially in saving running time, reducing bias and improving capability of balancing accuracy and sensitivity. We also introduce a new method to classify circular RNAs and report a potential case of chimeric circular RNA.

## Background

**Fig. 1:**
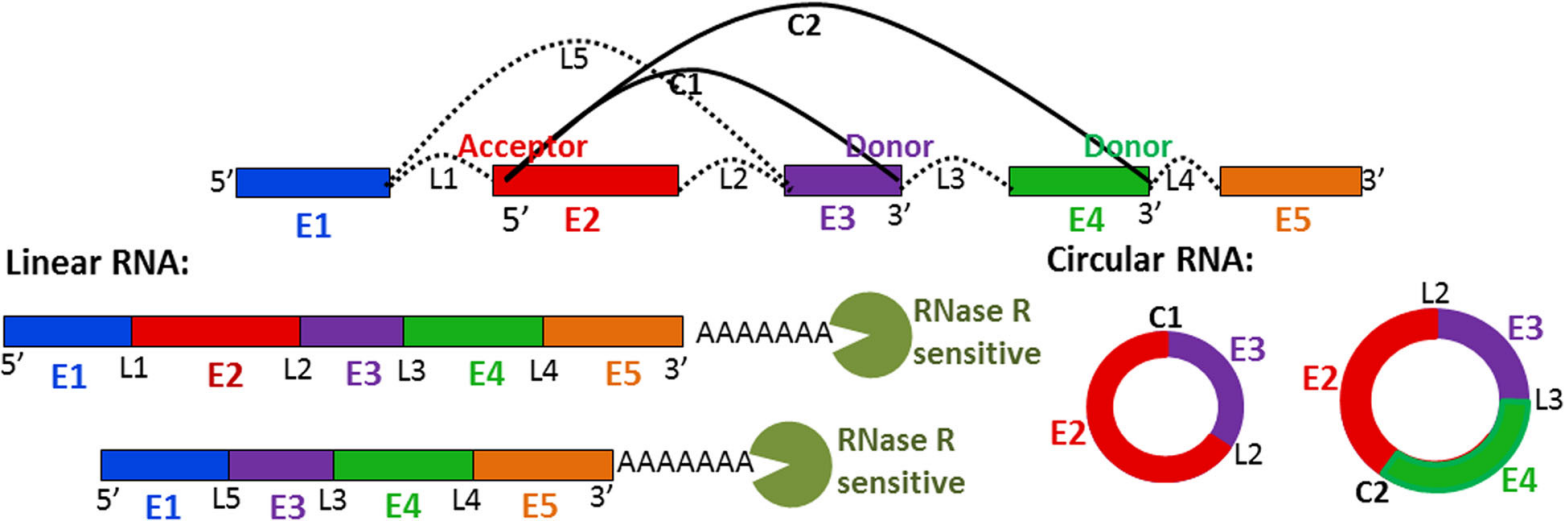
*E*_*i*_: exons, *L*_*i*_: linear splicing junction (for linear RNA). *C*_*i*_: circular splicing junction (for circRNA). Two sets of isoforms of linear RNA and circRNA are shown on left and right sides. 3^*`*^ in circular splicing junction is donor, while 5^*`*^ is acceptor. All linear isoforms are sensitive to RNase R. Circular isoforms show no significant decrease in abundance after RNase R treatment

Circular RNA, the RNA in a circular form through a usually 5’ to 3’ phosphodiester bond, is a type of non-coding RNA [1]. Circular RNA (or circRNA) is recently recognized as a new class of functional molecule [1]. CircRNA consists no 5’ or 3’ free terminus, as illustrated in Fig. 1, which makes it much more stable in the cells than linear RNA [1]. CircRNAs were originally thought as the byproduct from the process of mis-splicing, and considered to be of low abundance. Recently, however, the importance of circRNAs in gene regulation and their biological functions in some human diseases have started to be recognized [1–3]. Many of these circRNAs are stable and contain exons, but they are not translated into proteins [4].

There are two types of experimental methods currently that can be used to identify circular RNA [5]. First, since circRNAs lack a poly(A) tail [6], they can be retained in rRNA-depleted libraries by using expected depletion profile to assess results. Second, circRNA can be enriched in libraries treated with RNase R to digest linear RNA and make it easier to detect lowly expressed circRNA. With the high-throughput sequencing technologies, multiple bioinformatics tools have been developed recently for circRNAs detection from RNA sequence reads. Some of them require gene annotation while others do not. Those methods could be divided into two categories: (a) reads-mapping-based methods, such as CIRI [7]/CIRI2 [8], CIRCExplorer [9], Find-circ [10] and CircRNAFinder [11], and (b) k-mer-based methods, such as CircMarker [12].

Reads-mapping-based methods first map the RNA-seq reads onto a reference. For this purpose, CIRI uses BWA [13], while bowtie [14] and Tophat (TopHat-fusion) [15] are used by Find-circ and CIRCExplorer respectively. Since BWA and bowtie do not require annotations, all RNA reads need to be mapped to the entire reference genome. As CIRCExplorer, CircRNAFinder only performs reads mapping by using STAR in the range of annotated genomes as provided by annotation file. Those mapping methods have two major issues. First, reads-mapping-based tools are often computationally inefficient because mapping all reads can be slow, yet we note that many RNA-seq reads are irrelevant to circRNA detection. Second, these tools may miss circRNA in some cases due to errors in reads mapping [12].

Recently, we developed a k-mer-based tool called CircMarker [12], which uses an efficient k-mer table for circular RNA detection. Compared with the reads-mapping-based method, CircMarker has two major advantages. First, CircMarker looks for the circRNA-related reads for detection and does not depend on any third party mapping tool. Thus CircMarker is much faster than reads-mapping-based methods, especially for small data. Second, since the minimum comparison unit for CircMarker is a k-mer rather than reads, it can tolerate more errors and find more circular RNAs. However, CircMarker still has some issues. A key issue for CircMarker is the potential loss of information. CircMarker considers k-mers individually. That is, CircMarker does not consider the order of k-mers from either reads or exons, and this may lead to false positives when there are repetitive k-mers. Moreover, CircMarker becomes slow for large data.

In this paper, we present a new method named CircDBG for circular RNA detection with de Bruijn graph. Different from the normal de Bruijn graph, CircDBG uses it in a specialized way designed to call circular RNA, which is the first algorithm using de Bruijn graph for circular RNA detection. Through experiments based on simulated and real data, we demonstrate that this new method finds more reliable circRNA with low bias, runs faster and has better performance in balancing accuracy and sensitivity than existing methods.

Finally, we introduce a new method of classifying circular RNAs based on reads alignment and report a potential chimeric circular RNA that is found by CircDBG based on real sequence data.

## Method

### High-level approach

**Fig. 2:**
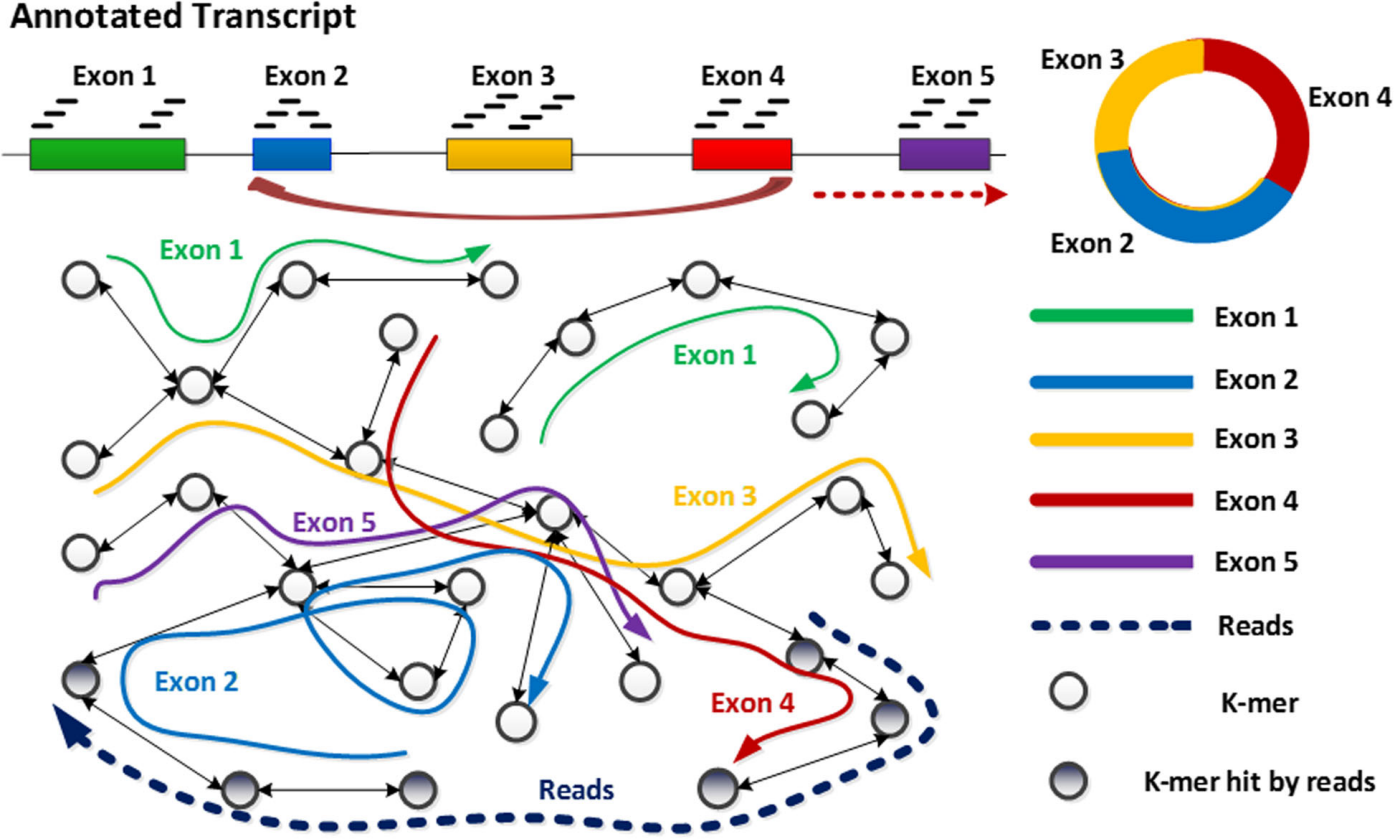
Five exons are in the genome (top left). Back splicing occurs from exon 4 to exon 2, which generates a circular RNA (top right). The bidirectional de Bruijn graph (bottom left) is built from the k-mers from each exon in the genome, where each exon is represented by the path with the same color in graph. The dotted line represents a RNA reads which supports the presence of circRNA case: the starting part of the read overlaps with the ending part of exon 4, and the ending part of the read overlaps with the starting part of exon 2

The key idea of CircDBG is creating a de Bruijn graph based on k-mers from the boundary parts of exons in annotated genome. As shown in Fig. 2, we take advantage of this graph to find the relationship between k-mer of reads and the potential donor/acceptor exon by tracking the path in the graph for circular RNA detection. Since the path provides a stronger signal for calling the two exons involved in the back splicing than individual k-mers, CircDBG can filter out more false positives than CircMarker. This is especially true when there are duplicate k-mers in exons and/or there are errors in the reads. To make CircDBG more efficient, we also develop various techniques.

### CircDBG

Our new CircDBG method contains three parts: (a) building de Bruijn graph, (b) finding potential donor/acceptor sites, and (c) detecting circular RNA. First, a memory-efficient de Bruijn graph is created, which records relevant information of annotated genome. Second, we filter out circRNA-related reads and find the potential donor/accepter exon. Finally, we compare k-mers from reads with the graph for circular RNA detection. Some parameters should be determined before running CircDBG, such as the length of k-mer. The maximum length of k-mers in CircDBG is 16, and we set 15 as its default value for all data analysis reported in this paper.

#### Build de Bruijn graph

We create de Bruijn graph for each chromosome separately, and use them in parallel with RNA sequence reads for circular RNA detection. All the k-mers used to create de Bruijn graph come from exon, and the k-mers from reads will be used to track the path in the graph for circular RNA detection. We use 2 bits to present each base in k-mer, and integer 32 is used to save the value of k-mer. Therefore, the maximum length of k-mer presented by each node in graph is 16 bps. For each chromosome, only the exons that contain back splicing signal (GT-AG) are considered. The exons with length shorter than the chosen k-mer length are ignored. Since the back-splicing only occurs near the boundary of exon, and one read cannot cover the whole exon, especially when the exon is very long, we only use k-mers near the boundaries of an exon when building the graph. The length of extraction is identified as:

$$ L_{seq} = L_{reads} - k - 5 $$

This means we require that there are at least 6 continuous k-mers should come from the other side of circular splicing junction. In another word, we require at least the length of *k*+5 in reads to come from the other site of circular RNA. If the length of an eligible exon is shorter than 2×*L*_*seq*_, the whole exon is used to build graph.

For example, if the length of reads and k-mer is 101 and 15 respectively, based on the equation, the length of extraction is 81, which contains 67 k-mers theoretically. Given an exon with the length of 1000 bps, two sequences will be extracted from the beginning part (1 to 81) and ending part (920 to 1000) respectively.

**Fig. 3:**
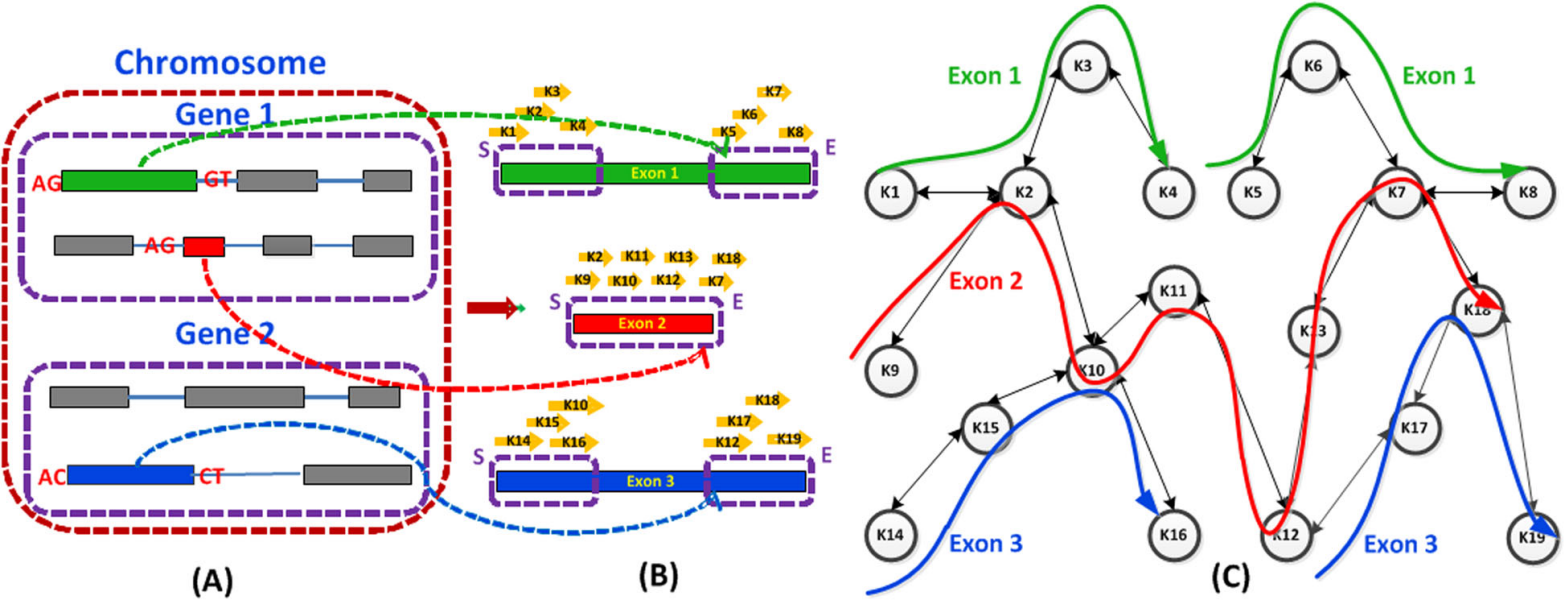
**a** Three exons with the back-splicing signal (AG-GT/AC-CT) are chosen. **b** The k-mers from the beginning part and ending part of exon 1 and exon 3 are collected, while all k-mers from exon 2 are selected since *Len*_*exon*2_<2×*L*_*seq*_. **c** The graph is constructed by these collected k-mers: exon 1/exon 3 are represented by 2 separate green/blue paths in the graph, and exon 2 is represented by one red path

All k-mers from the boundary parts of sequences are processed sequentially and converted into integers as the values of nodes in the graph. The edge of each node represents its next or previous neighbors. The procedure of creating de Bruijn graph is illustrated in Fig. 3. Since the node may be shared by multiple exons or appear multiple times in one exon, multiple groups of exon information are associated with each node. Each exon information contains the node’s position and multiple links. As same as the strategy of CircMarker, we use 5 values to represent the node position: one tag and four indexes (including chromosome, gene, transcript, and exon). Since the maximum index value (e.g. the number of exons in one transcript) is not large, we use light weight data structure to store those additional messages, such as exon index and transcription index, in each node to save the RAM[12]. The tag contains 4 different values: S/E and H/T, which specifies whether the k-mer comes from the starting or ending part and whether it is close to head or tail boundary of exon respectively. Since the closer a k-mer is to the back-splicing junction point, the higher possibility the k-mer can be contained by the supported reads, we call the node with tag H/T as premium node. The data structure discussed above can help to distinguish repetitive k-mers in the same exon or multiple exons, because it records all possible exon positions and the neighbors of each node. In addition, since we only extract k-mer from the boundary side of each exon, the number of collected k-mers is not large. With the help of additional messages recorded in each node, the maximum length of 16 bps is enough to distinguish the majority part of those k-mers. Here is an example of how we save the exon info for one node: suppose one k-mer is found in the valid part of exon 1 and exon 2, and it appears one time in exon 1 and two times in exon 2. Then, the k-mer is converted into an integer and set as the key of this node. Two exon information are associated with this node: exon 1 info contains node position and one link, while exon 2 info contains node position and two links. Each link includes the key of its previous and next node.

Finally, since the last node doesn’t have the next neighbor and the first node doesn’t have the previous neighbor, we set the value of this kind of next and previous neighbor node as 0 with tag “U”. These two special nodes indicate the ending and beginning of the path.

#### Find potential donor/acceptor sites

We first obtain the circRNA-related reads by using the similar strategy of CircMarker: filter out reads that none of sampled k-mers from the read has matches in the graph. Both original reads and its reverse complementary are considered. If the original reads is failed to be found back in graph, CircDBG will consider its reverse complementary.

In order to identify the back splicing of circRNAs, we need to search for the potential donor and acceptor sites. The donor side comes from the ending part of the exon, which is contributed by the starting part of the reads, while the acceptor side comes from the starting part of the exon, which is contributed by the ending part of the reads.

**Fig. 4:**
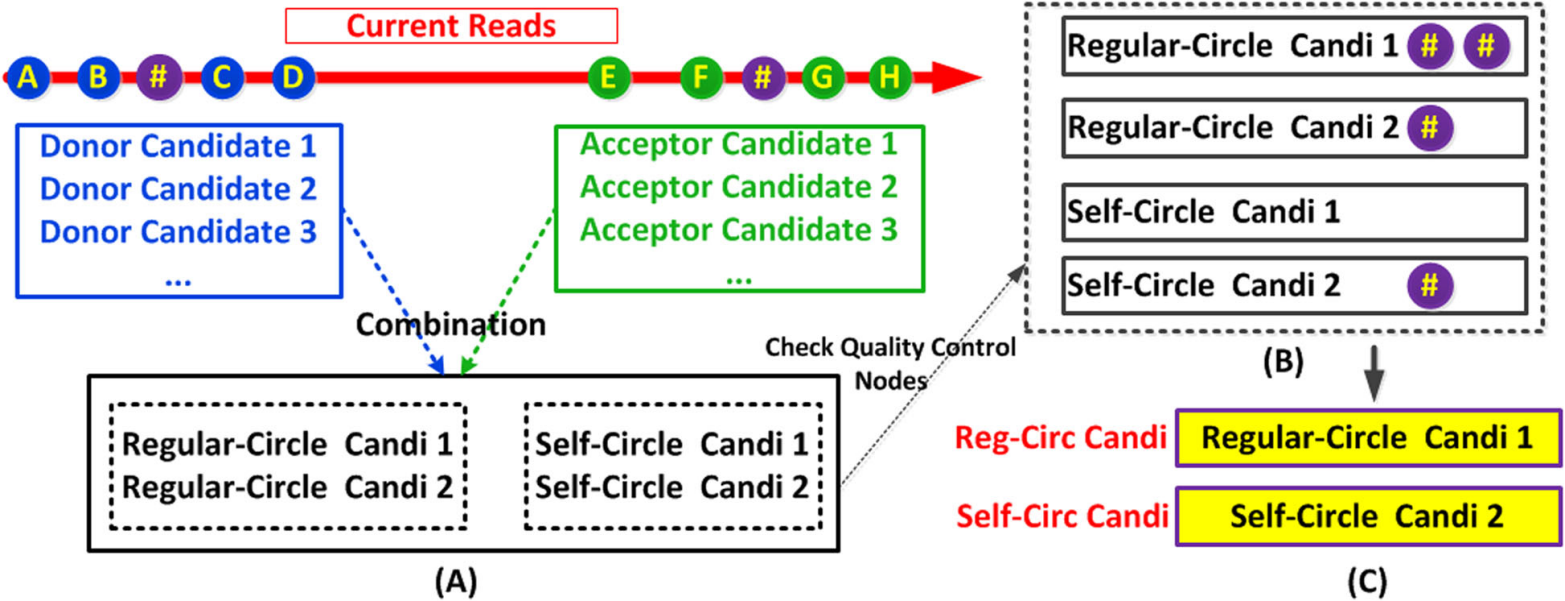
**a** Each node stands for k-mer. Four blue nodes represent the k-mers chosen from the head part of reads which are related to donor, while four green nodes represent the k-mers from the tail part which correspond to acceptor. Two purple nodes with symbol # represent the quality control nodes. Two self-circle cases and two regular-circle cases are found by the combination of donor and acceptor candidates. **b** Check whether or not quality control nodes support each circular case. **c** “Regular-Circle Candi 1” and “Self-Circle Candi 2” are kept, since they are supported by more quality control nodes than others in their case group respectively

To find potential donor candidates, we sample four k-mers from the beginning to the end of the reads, and search for each k-mer’s hit in the graph. A valid hit means the k-mer can be found in the graph and its next neighbor in graph can be found in the reads. The exon supported by at least two valid hits with tag T/E are collected as the donor candidate. For the potential acceptor candidates, we sample four k-mers from the end to the beginning of the reads, and apply the similar procedure as that of the donor candidate. There are two differences here: its previous neighbor is tracked and the valid hit should contain the tag H/S. We also collect two additional k-mers from reads for quality control. We try all combinations from the donor and acceptor candidates. If the donor and acceptor come from the same exon, we think this is the potential self-circle case. Otherwise, it belongs to regular-circle case if the donor and acceptor come from the same transcript in the order of back to front. If there are more than one candidate for each circle case, we only consider the candidate supported by the maximum number of quality control nodes. See Fig. 4 for an illustration. Note that for the regular-circle candidate, if donor and acceptor nearby each other with the same sequence value, they may be from genome repeats and this candidate is ignored.

#### Circular RNA detection

**Fig. 5:**
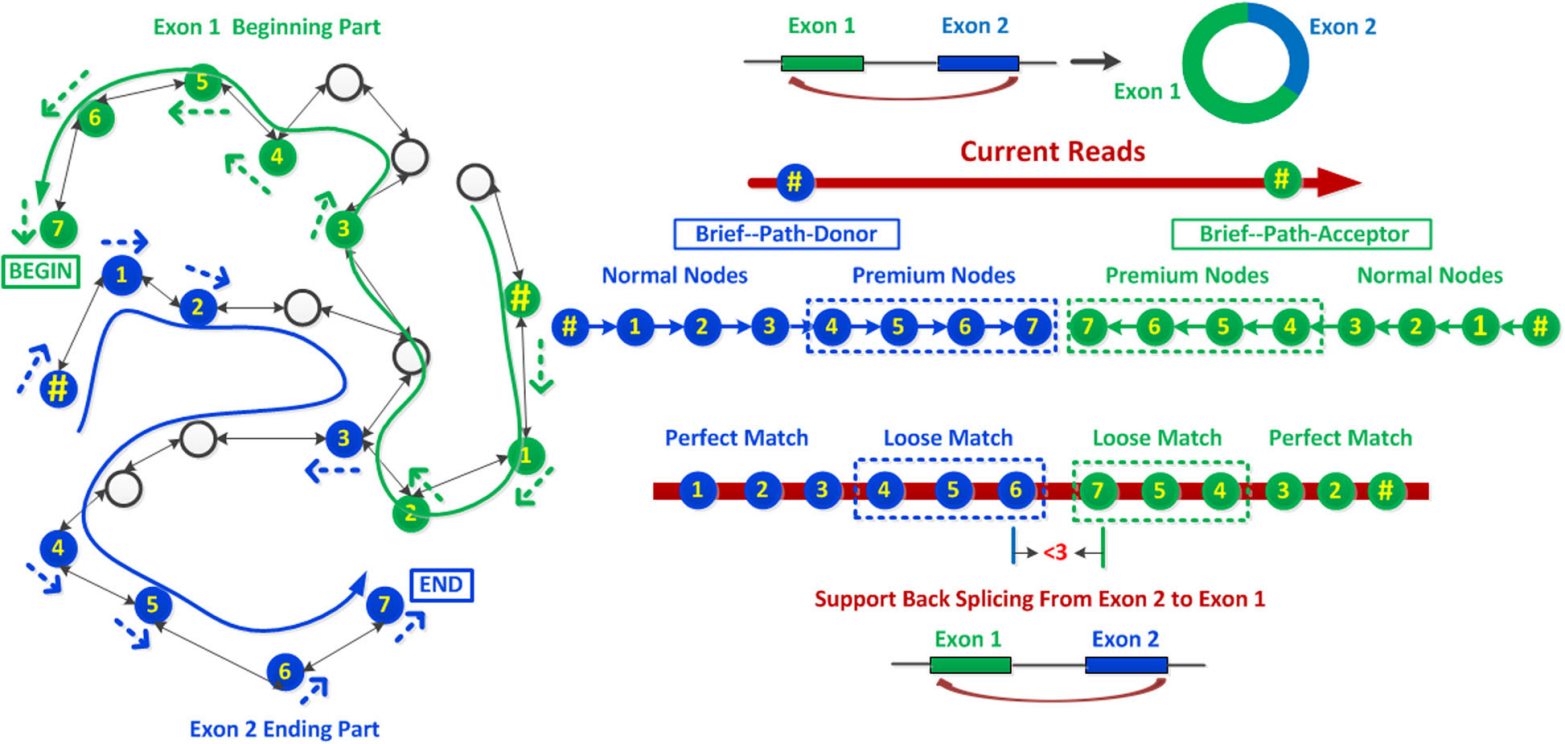
Back splicing occurs from Exon 2 to Exon 1 which generates a circle. The blue node with symbol # is the anchor node of donor and the blue path in graph represents the donor (Exon 2) by tracing backward from the anchor node. “Brief-path-donor” contains eight blue nodes in path, including four premium nodes. Green path in graph represents the acceptor (Exon 1) by tracing forward from anchor node. “Brief-path-acceptor” contains eight green nodes in path, including four premium nodes. Six nodes from the brief path of donor and acceptor can find hits in reads respectively. Since there are more than 70% hits and the distance between nodes 6 and 7 is shorter than 3 bps, this reads is considered to support the back splicing from Exon 2 to Exon 1

For each circRNA candidate, we try to find the first k-mer in the reads from the beginning to the end that can find hits in the graph with the same donor information identified in the current candidate. Then, we view this hitting node as an anchor and iteratively search for its next neighbor in the graph continuously. Once we get the path, we save a “brief-path-donor” by only keeping the first three nodes, the nodes with index divisible by 3 and the last node which contains the terminal signal in its next neighbor, as shown in Fig. 5. This brief path can speed up the later comparison while keeping the same accuracy. Here, when we check the full path in the graph, the search is terminated if the path is longer than the length of the reads. In addition, the candidate is ignored if the length of the full path is too short. Similar procedure is applied to extract the “brief-path-acceptor” by tracing the previous neighbor continuously from the anchor node, which is the first valid hit case with the same acceptor info from the end to the beginning of the reads. The total length of these two brief paths should be long enough or contain more than two premium nodes by each of them.

Once those two brief paths are prepared, we check if the nodes in “brief-path-donor” can find hits in the reads from the beginning to end sequentially. We perform “perfect match” for regular nodes. For the premium nodes, we perform“loose match”: the node sequence is divided into three parts and is viewed as a hit if at least two parts could be matched perfectly. If the number of hits is larger than our pre-defined threshold, we consider that the donor side is well supported by current reads. Otherwise, we apply a weak threshold if the hit nodes contain at least one premium node to guarantee the donor junction point to be covered by reads. The procedure of acceptor verification is similar to that of donor case. The only difference is that we try to check if the nodes in “brief-path-acceptor” can find hits in reads from the end to beginning sequentially. Then, we check the distance between two last mapping nodes in reads from those two brief paths respectively. The circRNA candidate is kept only if the distance is <3 bps. See Fig. 5 for an illustration.

Finally, we merge similar candidates that share similar boundary of both donor and acceptor site with maximum 8 bps differences by using the candidate with the shortest summary length of donor and acceptor to represent the final result.

## Results

Comparing different circRNA detection methods is not straightforward. The field lacks a gold standard for assessing the accuracy of their genome-wide predictions [5]. In addition, although several circular RNA databases have been released recently, such as circRNADb [16] and CircBase [17], the data in these databases come from published papers which are obtained from existing circRNA detection tools and only a few of those data have been verified through biological experiments. In this paper, we use four different strategies for evaluation. All of these strategies calculate the accuracy and sensitivity of each tool as follows, where *T* is the total called circRNAs by a tool, *T*_*hit*_ is the number of called circRNAs which find matches in the benchmark. “Benchmark” is prepared in different ways for each strategy.

$$\left\{\begin{array}{l} Accuracy = \frac{|T_{hit}|}{|T|},\ {where}\ T_{hit} = T \cap Benchmark \\ Sensitivity = \frac{|T_{hit}|}{|Benchmark|} \end{array}\right. $$

In the first strategy, we use simulated data for comparison where the simulated circRNAs are benchmark.

We choose public database circRNADb in the second strategy, and all records in database are viewed as reliable circRNA. There are two goals of this comparison. First, we want to examine how well the public database is supported by each tool. The larger coverage in database the results from a tool has, the better the tool can support the database. Second, we evaluate the bias of each tool by checking the overlap between the results of the current tool and others respectively. The larger overlap means the lower bias.

In the third one, for real data, if two different datasets come from the same tissue with different experimental libraries, the intersection between the results of those two datasets could be considered as the reliable results for each tool, and the circRNAs supported by at least two tools could be viewed as the benchmark.

In the last strategy, we use the intersection between the results of RNase R treated and untreated data to get the reliable results for each tool, and the circRNAs supported by at least 2 tools are viewed as benchmark. See Additional file 1 (A. Benchmark used for comparison) for details on benchmark.

Our experiments show that no single tool always has the highest accuracy and sensitivity. Thus, we focus on comparing the balance between those two indicators by using F1 score.

The F1 score is calculated by $2\times \frac {Precision \times Recall}{Precision + Recall}$. In our cases, since there is no true negatives, and all non-true positives are viewed as false positive, the precision and recall are equal to accuracy and sensitivity respectively. So the F1 score is equal to $2 \times \frac {Accuracy \times Sensitivity}{Accuracy+Sensitivity}$.

Since some tools depend on annotation files whereas some others don’t, and the majority part of back splicing comes from the exons in genome, we choose circRNAs with junction points identified by annotated genome for comparison.

The results of the second strategy are shown in Additional file 1 (B. Real data: circRNADb with tissue H9 hESCs). CircDBG finds more circRNAs recorded in database than other tools, and it always gets the largest coverage (20 of 23) in each chromosome respectively. Moreover, CircDBG is the best tool with the lowest bias (contain majority results of other tools) and the fastest running time. The results of other three strategies are presented below.

### Simulated data

We use the latest simulator released by CIRI2 [8] to simulate circRNAs and RNA-seq reads. The length of simulated paired-end reads is 101 bps, and the coverages of circRNA and linear RNA are 10x and 80x respectively. The error rate is 1%. The major/minor normal distribution insertion length is 320/550, and the percentage of splicing for skipping exon is 40%. The reference and annotation file come from human chromosome 1 (GRCh37, version 18).

The simulated paired-end reads contain 1,115,738 pairs. 295 circular RNAs are simulated as benchmark. Accuracy, sensitivity and F1 scores are calculated for each tool.

**Fig. 6:**
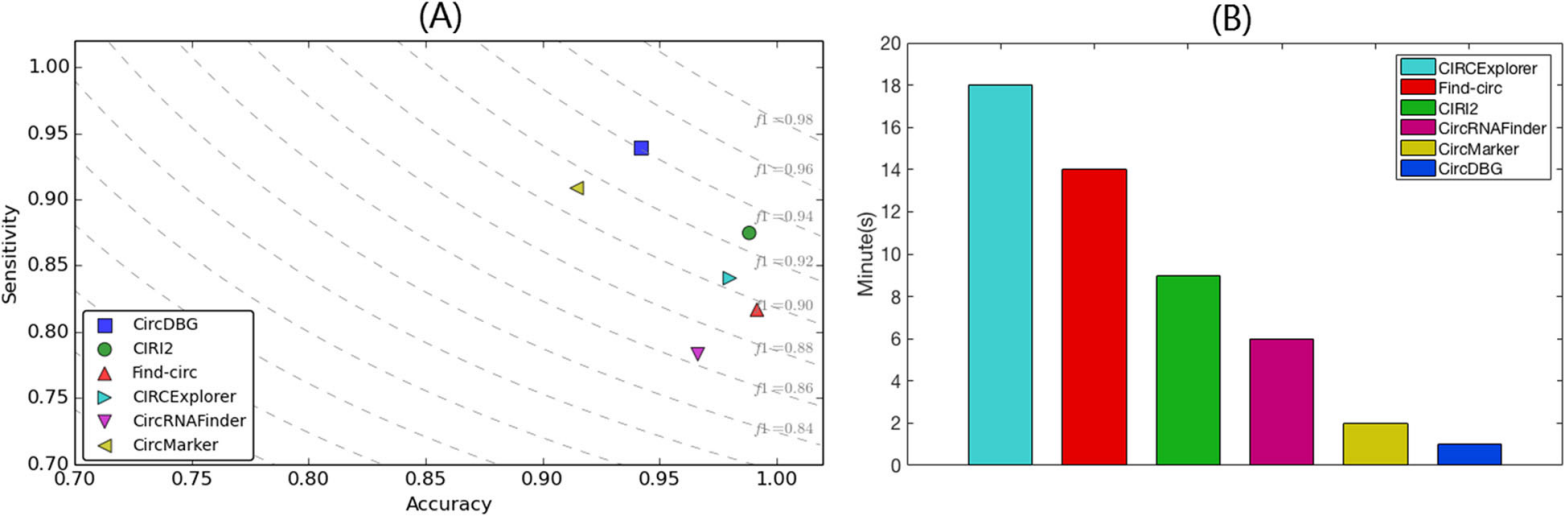
F1 score and running time on human simulated data **a** F1 score. Dotted line represents a fixed F1 score. The closer to top right, the higher F1 score it is. **b** Running time (in minutes)

As shown in Fig. 6, both accuracy and sensitivity of CircDBG are around 94%, and it gets the highest F1 score (0.9406). This means that CircDBG is the best tool for balancing accuracy and sensitivity. CircDBG also has the fastest running time.

### Real data: two different prepared libraries from same tissue

This comparison is based on two different libraries from the same issue. Intuitively, true circRNAs should be called for both libraries. We get the reliable results for each tool by taking the intersection between two differently prepared libraries from the same tissue. The results that are supported by both libraries are viewed as reliable. RNA-seq reads SRR4095542 and SRR5133906 are used for data analysis. The first library (43,488,788 paired-end reads) is prepared by 3 glioma and paired normal brain tissue. For the second library (54,732,199 paired-end reads), ten human glioblastoma samples are mixed as tumor group, and their periphery normal tissues are mixed as control group. Total RNAs in the second library are extracted and treated with RNase R to remove the linear RNAs.

The length of reads is 150 bp in both libraries. Recall that the intersection of the called circRNAs from two libraries is used as the final result for each tool, and circRNAs supported by at least 2 tools are viewed as benchmark.

**Fig. 7:**
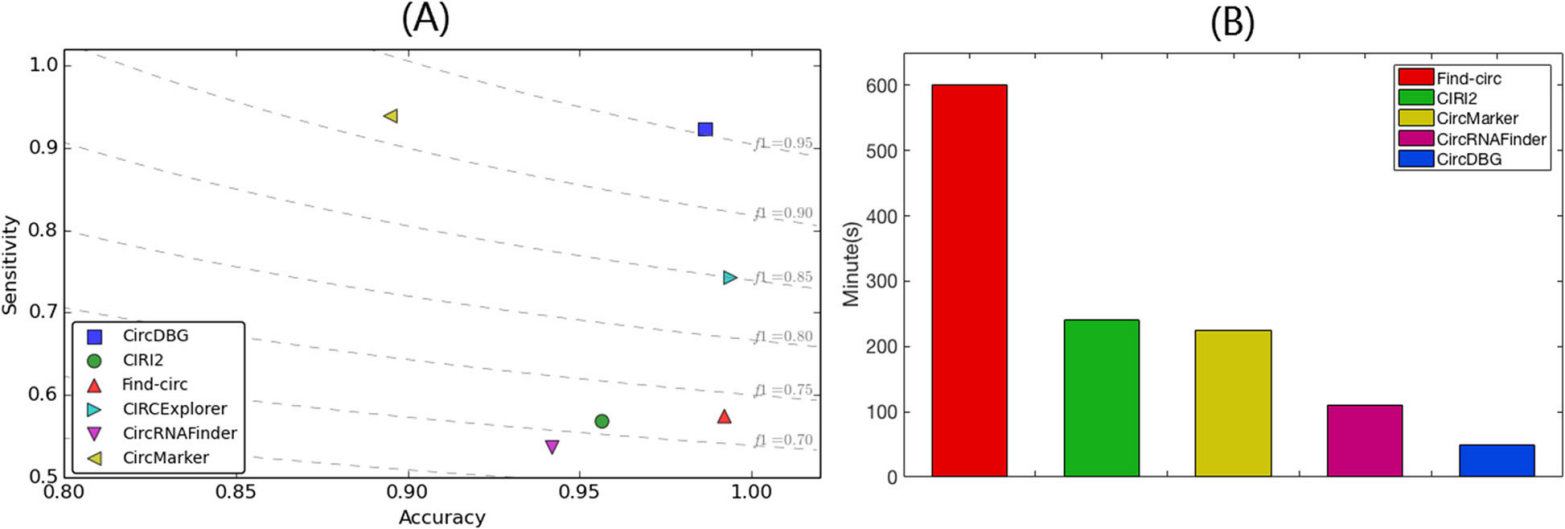
F1 score and running time on human glioblastoma samples **a** F1 score. Dotted line represents a fixed F1 score. The closer to top right, the higher F1 score it is. **b** CIRCExplorer takes more than 36 hrs and is not shown here

Our results are shown in Fig. 7. CircDBG has the highest F1 score (0.9539). In addition, the accuracy of CircDBG is 98.65% with the highest sensitivity and the fastest running time.

### Real data: RNase R treated and untreated samples

This comparison is performed with RNase R treated and untreated libraries. We collect two sets of treated and untreated reads from homo sapiens and mus musculus respectively. A circRNA is viewed as reliable if it can be found by both RNase R treated and untreated reads (linear RNAs tend to degrade by RNase R treatment). SRR1636985 (treated, 13,309,745 paired-end reads) and SRR1637089 (untreated, 44,933,450 paired-end reads) from HeLa cells are used for human.

The length of reads in both libraries is 101 bps. For mouse libraries, SRR2219951 (treated, 22,330,976 paired-end reads) and SRR2185851 (untreated, 32,939,809 paired-end reads) are selected, which are prepared by mouse brain at the age of 8 to 9 weeks.

The length of reads in the two groups varies with the maximum 100 bps. For each species, we obtain final results for each tool by taking the intersection between the results based on treated and untreated reads and build the benchmark by choosing the circRNAs supported by at least two tools.

**Fig. 8:**
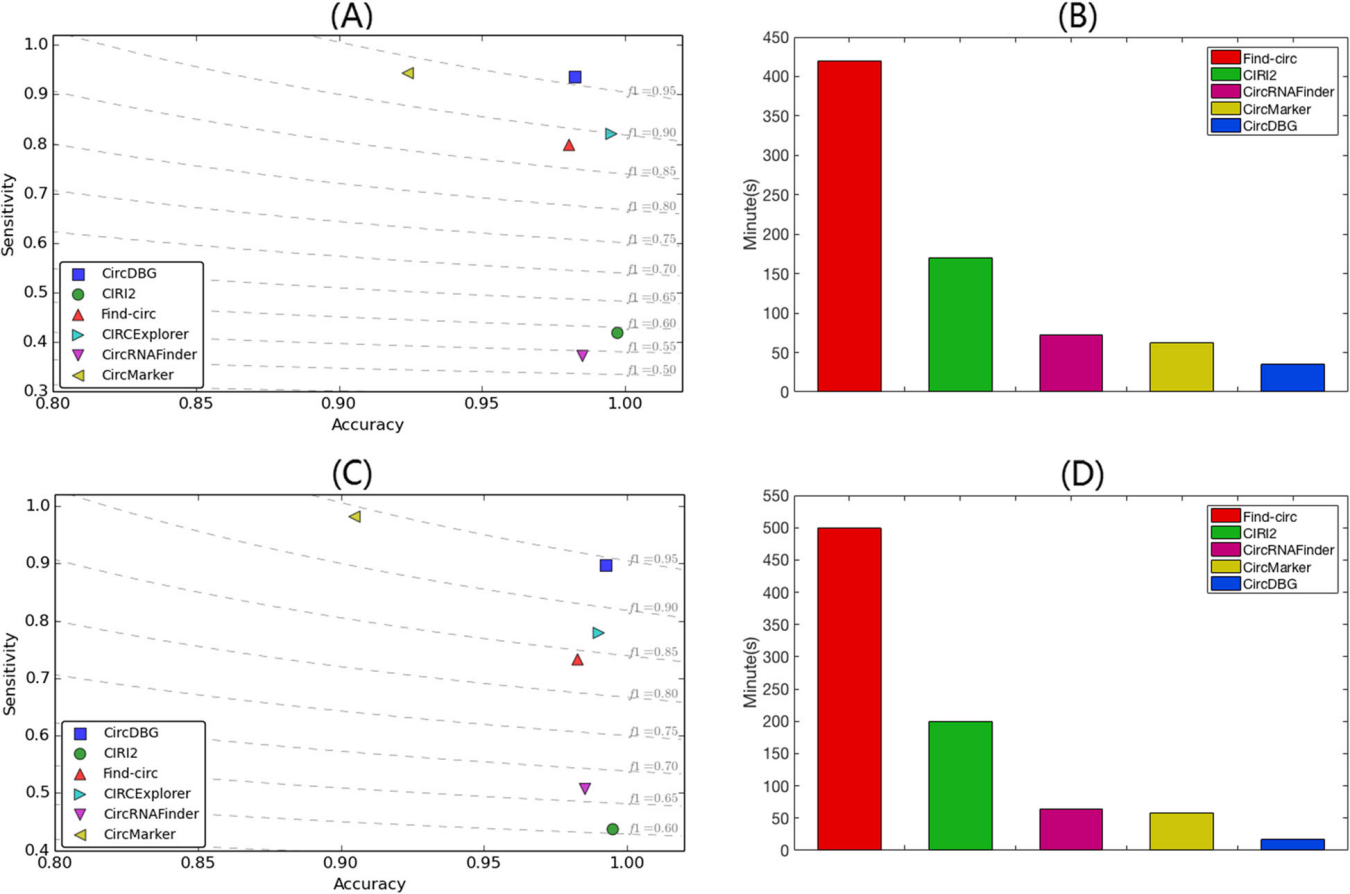
F1 score and running time on human and mouse samples **a** F1 score on human HEK293 real data **b** Running time on human HEK293 real data. **c** F1 score on Mus Musculus real data and **d** Running time on Mus Musculus real data. CIRCExplorer takes 15 hrs and 21 hrs for human and mouse respectively, and is not shown in **b** and **d**

As shown in Fig. 8, CircDBG has the highest F1 score and the fastest running time in both human (F1 Score: 0.9589) and mus musculus (F1 Score: 0.9424). The accuracy of CircDBG in human and mouse are 98.24% and 99.25% respectively. CircDBG and CircMarker are the top two tools that get the highest sensitivity in both human and mouse libraries.

### Case study: chimeric circular RNA

We develop a novel evaluation scheme, which analyzes what reads contribute to the calling of circRNAs. In this scheme, CircRNA reference is generated by linking the ending part of donor exon with starting part of acceptor, and the circRNAs detected by CircDBG are classified into 5 categories based on the alignment result of reads. See Additional file 1 (C. Classification of circRNA by reads) for details.

**Fig. 9:**
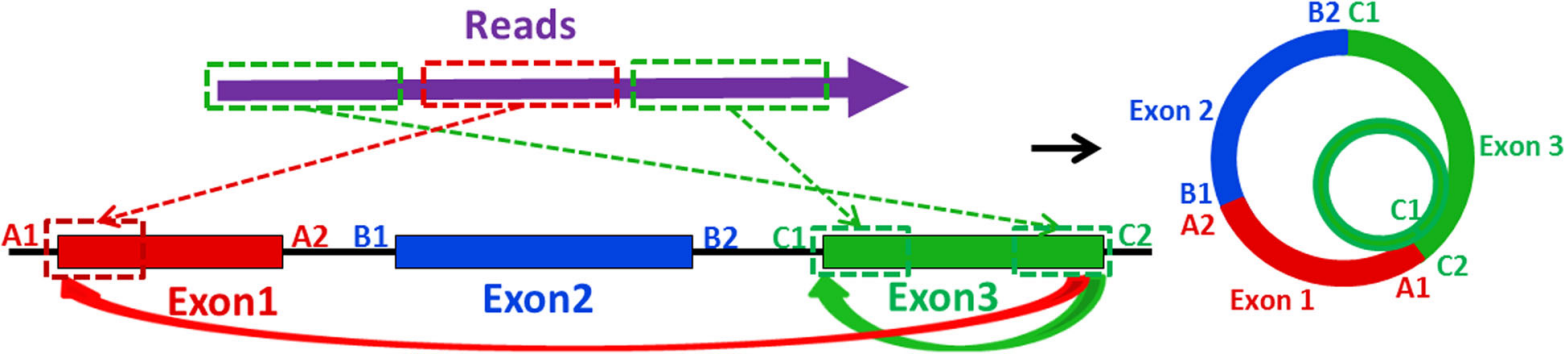
The first and the second part of reads support the regular-circle RNA case from exon 3 back to exon 1, while the first and the third part of reads support the self-circle RNA case of exon 3

We notice that there is a special case when classifying the detected circRNA with real data: some reads not only support regular-circle case, but also support the self-circle case for the exon contained by current regular-circle case, which is illustrated in Fig. 9. This case may relate to chimeric phenomenon in circular RNA which can be comprehended as “circle in circle”.

We collect this type of chimeric circular RNA in real data and find this phenomenon exists in all real data we analyze. For example, in H9 hESCs, 102 chimeric cases can be found among the total 11,931 circular RNA. Although the percentage seems to be small, the number of the chimeric cases is significant.

## Discussion

CircDBG uses a specialized designed de Bruijn graph to call circular RNA. First of all, the de Bruijn Graph designed for regular usage, such as assembly, only contains limited information. Differently, the graph created by CircDBG contains a bunch of additional messages, such as genome ID, transcript ID, its neighbors and part tag. Specifically, multiple groups of those messages will be recorded if the k-mer appeared in multiple different exons or multiple different positions in one exon. Secondly, in order to speed up the whole algorithm, CircDBG does not use all k-mers from exons to create the graph, and the unordered map is applied to store the graph, which is different from other methods. Finally, CircDBG does not scan the whole graph but only tracks the interesting path to find potential circular RNA.

The literature of the algorithm on creating de Bruijn graph is broad [18–21], however, it is hard to compare the performance of de Bruijn graph created by CircDBG separately with other state-of-art methods. This is because the purpose of our de Bruijn graph is to find circular RNA rather than doing normal assembly. Therefore, the de Bruijn graph needs to be designed and used in a different way as we mentioned above to make it compatible with the whole algorithm for circular RNA detection. The high efficiency data structure introduced by other state-of-art methods cannot be applied to detect circular RNA directly. In addition, since CircDBG is the first algorithm that uses de Bruijn graph for circular RNA detection, we do not compare the performance of de Bruijn graph separately with others. Moreover, since our purpose is to find circular RNA, we only compare the performance of the whole algorithm with other similar methods for circular RNA detection. Based on comparison results, the de Bruijn graph created by CircDBG could let the whole algorithm perform better than others.

Regarding time consumption, CircDBG belongs to k-mer-based method. Generally speaking, k-mer-based tools perform better than reads-mapping-based tools. This is because reads-mapping-based tools are often computationally inefficient, since mapping all reads can be slow and many RNA-seq reads are irrelevant to circRNA. Differently k-mer-based tools only look for the circRNA-related reads for detection, and they do not depend on any third-party mapping tool. Compared with the existing k-mer-based methods, CircDBG also outperforms others. This is because the existing tools scan each k-mer of the reads to extract their affiliated messages from k-mer table, while circDBG only scan some of k-mers sequentially from the reads by taking advantage of “brief-path-donor” and “brief-path-acceptor” to find the qualified paths of circRNA, which is much faster and more reliable.

CircDBG sets 5 and 16 as the number of continuous k-mers and the length of k-mer (as shown in equation 1) respectively by default. Theoretically, we can increase or decrease the number of continuous k-mers. If this value is increased, some of circular RNA, which are supported by the short part of reads (imbalance cases), may be missing. If this value is decreased, we may gain some false positives. Based on our empirical study, “K+5” (6 k-mers) could give us the reasonable results. As similar as the number of continuous k-mers, the length of k-mer can also be changed. If the length is increased, some reads that do not match well with the donor/acceptor site of circular RNA may be discarded because the capability of error tolerance turns to be weak. If the length is decreased, it may cause a large number of unexpected repeats, which makes it’s hard for us to identify the valid path in graph. Based on our testing, 15 bps length of k-mer could give us the reasonable result.

Since all of circular RNAs that contain the exon shorter than k-mer will be ignored by CircDBG, we calculate two statistic results, including the number of exon shorter than k-mer and the number of circular RNA called by different algorithms that contains the exon shorter than k-mer. The sample comes from RNase R treated real data of HeLa cells (SRR1636985). The default length of k-mer is 15 bps. The results show that there are total 612294 exons contained by annotation file (homo sapiens GRCh37.75), among which 3652 exons are shorter than 15 bps. 12533 circular RNA are detected in total by the tools that we used for comparison, and 6 circular RNA contain the exon shorter than 15 bps.

Most of reads-mapping-based circular RNA detection methods are pipeline, which require other third-party tools such as BWA, Bowtie and Tophat-fusion. As a result, the RAM usage for those methods is scattered. Generally speaking, the k-mer-based methods, such as CircMarker and CircDBG, consume more memory than mapping-based approaches. However, since high-performance computing (HPC) is wildly used to run the software in the research field of bioinformatics, the size of RAM that we can use in HPC is much larger than personal computer. For our testing cases, we run CircDBG in one computing node of HPC, and the memory size of each computing node is 128 GB. The peak RAM costs of CIRC2, Find-circ, CIRCExplorer, CircRNAFinder, CircDBG and CircMarker are around 10.4*%*, 2.1*%*, 12.8*%*, 34.1*%*, 13.3*%* and 12.7*%* respectively for the whole-genome analysis based on RNase R treated sample of HeLa cells (SRR1636985, 13,309,745 paired-end reads). Since the size of RAM in HPC is much larger than what we need, sacrificing RAM to gain better performance of analysis is reasonable.

Finally, since both CircDBG and CircMarker belong to k-mer-based method, there is an example to demonstrate CircDBG could avoid some false positives compare with CircMarker. Suppose we have an exon and a read as below, and the length of k-mer is 5. Therefore, the total number of k-mer in reads is 20, and the k-mer “GTGAT” repeats three times.

$$ {\begin{aligned} \left\{\begin{array}{l} Exon: GTGATATGTGGGGTTGGTGATTTTCTCTGTGATCAGTGATGGG \\ Reads:GTGATATGGTGATGGGGTGATTTT \end{array}\right. \end{aligned}}  $$

For CircDBG, the maximum length of detected valid path is 4, which means the maximum number of continuous k-mer in exon detected by CircDBG is 4 (“GTGAT”, “TGATA”, “GATAT” and “ATATG”). For CircMarker, since we only consider how many k-mers of reads could be found back in exon, the number of valid k-mer is 17, which means there are 17 k-mers in reads could be found back in exon. Since the matching status calculated by CircDBG is 4 out of 20, which is too low, this matching case (potential site of circular RNA) will be discarded by CircDBG. This is correct because this read does not well match the given exon. However, since the matching status calculated by CircMarker is 17 out of 20, which means most of k-mers from reads (without consider their orders) could be found back in exon, CircMarker views this read as the strong supporter of this exon, which is incorrect.

## Conclusion

In this paper, we develop a new method called CircDBG for circular RNA detection, which is based on de Bruijn graph. The graph represents the relationship between k-mers in original exon and reads. This contributes to more accurate results and runs much faster compared with the existing k-mer-based methods. CircDBG is the stand alone tool and does not depend on any other third party tools. CircDBG can be downloaded from: https://github.com/lxwgcool/CircDBG.

## Supplementary information

**Additional file 1** Supplemental materials for “Detecting Circular RNA from High-throughput Sequence Data with de Bruijn Graph”.

## Data Availability

The software is available under the GPLv3 licence at https://github.com/lxwgcool/CircDBG. The sample data sets used in this paper are available at European Nucleotide Archive (https://www.ebi.ac.uk/ena) with identification numbers SRR4095542, SRR5133906, SRR1636985, SRR1637089, SRR2219951, SRR2185851 and SRR901967. The circular RNA database used in this paper is available at circRNADb (http://202.195.183.4:8000/circrnadb/circRNADb.php). The reference data and annotation files used in this paper are available at ensembl (http://useast.ensembl.org/index.html).

## Abbreviations

BWA: Burrows-Wheeler aligner
CircRNA: Circular RNA
HPC: High-performance computing
RAM: Random access memory
RNA: Ribonucleic acid
