# Supplementary material for: Detecting circular RNA from high-throughput sequence data with de Bruijn graph
Source: BMC Genomics. 2020 Mar 5;21(Suppl 1):749. doi: 10.1186/s12864-019-6154-7 (PMC7057571; doi:10.1186/s12864-019-6154-7)
Supplement: Supplementary file 1 — Additional file 1 Supplemental materials for “Detecting Circular RNA from High-throughput Sequence Data with de Bruijn Graph”. [file 12864_2019_6154_MOESM1_ESM.pdf]

Supplemental materials for "Detecting Circular RNA from High-throughput Sequence Data with de Bruijn Graph"

A Benchmark used for comparison

The benchmark is represented by the formula below, where T is the final result of current tool, which comes from the intersection between the detection results of dataset A (e.g. treated) and B (e.g. untreated). "n" is the total number of tools, and the final benchmark contains all the results which are supported by at least two tools.

$$Benchmark = \bigcup_{i,j=\binom{2}{n}} T_i \cap T_j, T = T_A \cap T_B$$

B Real data: circRNADb with tissue H9 hESCs

We choose the database circRNADb in this comparison, and all records in database are viewed as reliable circRNA, since these records were collected from several published studies and only the circRNAs supported by more than one read are recorded [1]. There are two goals of this comparison. First, we want to examine how well the public database is supported by each tool. The larger coverage in database the results from a tool has, the better the tool can support the database. Second, we evaluate the bias of each tool by checking the overlap between the results of the current tool and others respectively. The larger overlap means the lower bias. All circRNAs recorded in circRNdb come from Homo Sapiens, and there are total 10,631 circular RNAs from H9 hESCs, which are used for comparison. The real reads SRR901967 are chosen for data analysis. These reads are specially designed for examining circular RNAs in H9 human embryonic stem cells with RNase R treated. It contains 41,342,095 single reads with the length 100 bps. We use three statistics in the comparison, including the number of circular RNAs hitting database (including the hitting situation in each chromosome respectively), the overlap between the results of each tool, and the running time.

Our results are shown in Figure S1. CircDBG covers more circular RNAs recorded in database than other tools, and it always gets the largest coverage (20 of 23) in each chromosome respectively. In addition, CircDBG and CircMarker overlap with more results from other tools. Moreover, CircDBG performs better than CircMarker in overlapping with two tools (CIRI2 and CIRCExplorer) and similarly in overlapping with other two tools (Find-circ and CircRNAFinder), which means CircDBG is the best tool with the lowest bias. Finally, CircDBG is much faster than other tools.

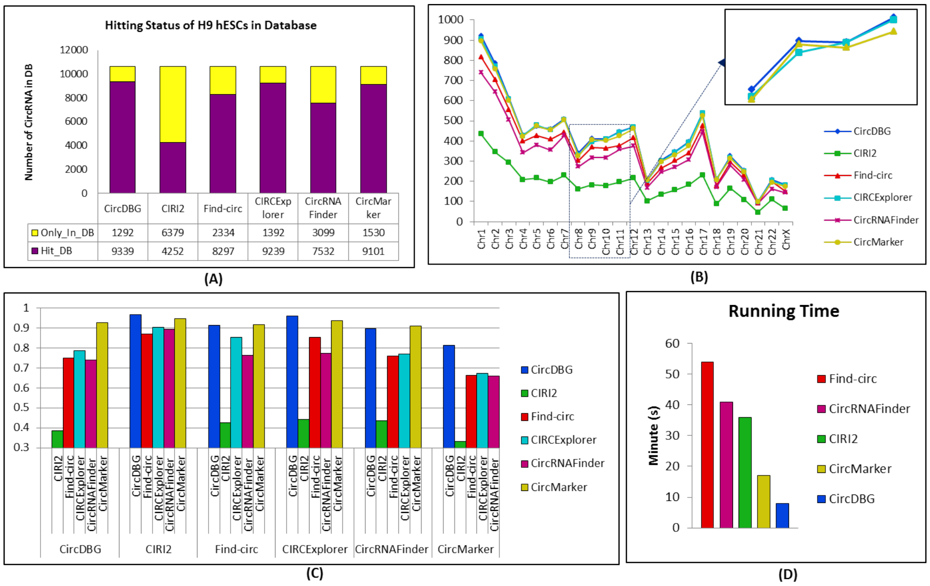

Fig. S1. (A) The yellow bar represents the circRNA from H9 hESCs recorded in database but not contained by the results of current tool, while the purple bar means the circRNA contained by both of them. (B) The plots represent circRNA contained by the current tool and database in each chromosome. (C) There are 6 groups, and the bars in each group mean the number of results of current tool covered by other tools. (D) CIRCExplorer takes more than 15h and is not shown here.

C Classification of circRNA by reads

We note that there are some differences among the results of different tools. In this section, we take a closer look at the results in order to see if there are some specific groups of circRNAs that can only be found by CircDBG.

Since each circular back splicing is detected by reads, we want to find out how those reads contribute to circRNA detection. Intuitively, if the error rate of reads is high, this reads may not be used for detection by some tools. In addition, if the part of a read which supports donor or acceptor is too short, the read may be ignored by some tools as well. Moreover, if one part of reads doesn't match either donor or accepter, it may also be discarded by some tools. Now, we align circRNA with their supported reads by BLAST. Five different categories are identified, including LowQuality, Imbalance, AdditionalPart, Bad and Good. "LowQuality" means the lowest error ratio is larger than 1% for all supported reads. "Imbalance" means the longest alignment segment of either the donor part or the acceptor part is shorter than 25 bps. If the minimum difference between alignment and reads length is larger than 10 bps, it is considered to be "AddionalPart". "Bad" means the junction point is out of the alignment range

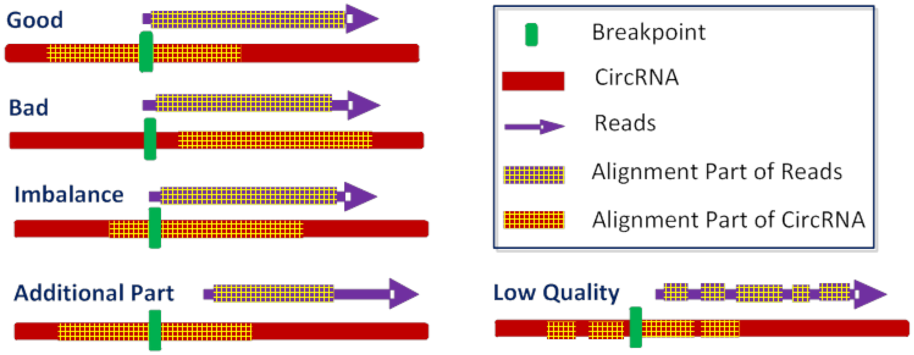

Fig. S2. circRNA reference (Red Bar) is generated by linking the ending part of donor exon with starting part of acceptor, and green bar is used to represent the circular junction point. "Good" means the most part of reads can be aligned to circRNA, and the length of alignment part in either side of junction point is $>$ 25 bps. "Bad" means the junction point is out of alignment part in circRNA. "Imbalance" means the alignment part on one side of junction point is too short. "Additional Part" means a continuous part of reads fails to align to circRNA reference. "Low Quality" means some gaps appear in alignment results.

for all supported reads. Otherwise, the quality of current circRNA is set as "Good". All of these five categories are illustrated in Figure S2.

Since CircDBG outputs the support reads of each detected circRNA, we apply this classification strategy to the called circRNAs by CircDBG in the real data. We find circRNA in "Good" category could be detected by most existing tools. However, the majority of circRNAs in "Imbalanced", "Additional part" and "Low Quality" categories are only detected by CircDBG and CircMarker. For example, in H9 hESCs, the detected number of circRNA for the categories of "Good", "Bad", "Additional Part", "Imbalance" and "LowQuality" are 8,604, 1, 654, 1, 2,532 and 139, and less than 30% of "Additional Part", "Imbalance" and "LowQuality" can be found in the results of reads-mapping-based methods. The tool named "CircAssistant" is developed to make this classification and to report the chimeric circular case based on the result of CircDBG. It could be downloaded from https://github.com/lxwgcool/CircDBG/Circ_Assistant.

D Command lines used in 6 tools for circRNAs detection

CircDBG

Key parameters set in configure file: KmerLen=15, KmerRatio=60
$CircDBG ./config.ini

CIRI2

$BWA index -a bwtsw $REF
$BWA mem -a -D 0 -t 24 ../chr1.fa ../PE1.fq ../PE2.fq > ./ciri_reads.sam
$perl $CIRIEXE -I $SAMFILE -O ../CIRI_Result -F $REF -A $GTF -T 24

Find-circ

$bowtie2-build $REF $REFFOLDER/REFINDEX

$bowtie2 p24 –very-sensitive –score-min=C,-15,0 –mm -x $REFFOLDER/REFINDEX -q $READS 2 > bowtie2.log — samtools view -hbuS - — samtools sort - test_vs_REFINDEX

$samtools view -hf 4 test_vs_REFINDEX.bam — samtools view -Sb - > unmapped_REFINDEX_test.bam

$EXEROOT/unmapped2anchors.py unmapped_REFINDEX_test.bam ¿ anchors_REFINDEX_test.fastq

$mkdir -p REFINDEX_test_out

$bowtie2 -q -U anchors_REFINDEX_test.fastq -x $REFFOLDER/REFINDEX –reorder –mm –very-sensitive –score-min=C,-15,0 2 > bt2_secondpass.log — $EXEROOT/find_circ.py -G $REF -n test -p REFINDEX_test_ –stats REFINDEX_test_out/sites.log –reads REFINDEX_test_out/spliced_reads.fa > REFINDEX_test_out/splice_sites.bed

$grep CIRCULAR ./REFINDEX_test_out/splice_sites.bed — grep -v chrM — grep UNAMBIGUOUS_BP — grep ANCHOR_UNIQUE — $EXEROOT/maxlength.py 100000 > ./REFINDEX_test_out/circ_candidates.bed

CIRCExplorer

$bowtie2-build ../../Tophat/REFINDEX.fa REFINDEX

$tophat2 -a 6 –microexon-search -m 2 -p 16 -G $GTF -o tophat $REFFOLDER/REFINDEX $READS

$BEDTOOLS/bamToFastq -i ./tophat/unmapped.bam -fq ./tophat/unmapped.fastq

$tophat2 -o tophat_fusion -p 16 –fusion-search –keep-fasta-order –no-coverage-search -G $GTF $REFFOLDER/REFINDEX ./tophat/unmapped.fastq

$GTF2GENEPRED -genePredExt -ignoreGroupsWithoutExons $GTF tmp

$awk 'BEGIN{FS="\t"};print $12"\t"$1"\t"$2"\t"$3"\t"$4"\t"$5"\t"$6"\t"$7"\t"$8"\t"$9"\t"$10' tmp > ./annotation.refFlat

$CIRCexplorer.py -f ./tophat_fusion/accepted_hits.bam -g /$REF -r ./annotation.refFlat

CircRNAFinder

$STAR –runThreadN 24 –runMode genomeGenerate –genomeDir $GenomeDir –genomeFastaFiles $Ref –sjdbGTFfile $GTF –sjdbOverhang 100

$runStar.pl $READS1 $READS2 $GenomeDir $OUTPUT prefix_whole_

$postProcessStarAlignment.pl $OUTPUT $RESULT

CircMarker

Key parameters set in Configure File: MinSupportReads=1, MaxSupportReads=999, KmerRatio=30, KMERLEN=15

$CircMarker ./config.ini

References

1. Chen, X., Han, P., Zhou, T., Guo, X., Song, X., Li, Y.: circrnadb: a comprehensive database for human circular rnas with protein-coding annotations. Scientific reports **6** (2016) 34985
